# Common and distinct patterns of brain activity alterations during inhibitory control in depression and psychostimulant users: a comparative meta-analysis of task-based fMRI studies

**DOI:** 10.1017/S0033291725101141

**Published:** 2025-07-28

**Authors:** Yuanyuan Li, Xiqin Liu, Jianyu Li, Qian Zhang, Nanfang Pan, Kui Luo, Graham J. Kemp, Qiyong Gong

**Affiliations:** 1 Department of Radiology, Huaxi MR Research Center (HMRRC), Functional and Molecular Imaging Key Laboratory of Sichuan Province, Frontiers Science Center for Disease-related Molecular Network, West China Hospital of Sichuan University, Chengdu, Sichuan, China; 2 Xiamen Key Laboratory of Psychoradiology and Neuromodulation, Department of Radiology, West China Xiamen Hospital of Sichuan University, Xiamen, China; 3 Research Unit of Psychoradiology, Chinese Academy of Medical Sciences, Chengdu, China; 4 Liverpool Magnetic Resonance Imaging Centre (LiMRIC) and Institute of Life Course and Medical Sciences, University of Liverpool, Liverpool, UK

**Keywords:** functional magnetic resonance imaging, inhibitory control, major depressive disorder, psychoradiology, psychostimulant use disorder

## Abstract

**Background:**

Major depressive disorder (MDD) and psychostimulant use disorder (PUD) are common, disabling psychopathologies that pose a major public health burden. They share a common behavioral phenotype: deficits in inhibitory control (IC). However, whether this is underpinned by shared neurobiology remains unclear. In this meta-analytic study, we aimed to define and compare brain functional alterations during IC tasks in MDD and PUD.

**Methods:**

We conducted a systematic literature search on IC task-based functional magnetic resonance imaging studies in MDD and PUD (cocaine or methamphetamine use disorder) in PubMed, Web of Science, and Scopus. We performed a quantitative meta-analysis using seed-based d mapping to define common and distinct neurofunctional abnormalities.

**Results:**

We identified 14 studies comparing IC-related brain activation in a total of 340 MDD patients with 303 healthy controls (HCs), and 11 studies comparing 258 PUD patients with 273 HCs. MDD showed disorder-differentiating hypoactivation during IC tasks in the median cingulate/paracingulate gyri relative to PUD and HC, whereas PUD showed disorder-differentiating hypoactivation relative to MDD and HC in the bilateral inferior parietal lobule. In conjunction analysis, hypoactivation in the right inferior/middle frontal gyrus was common to both MDD and PUD.

**Conclusions:**

The transdiagnostic neurofunctional alterations in prefrontal cognitive control regions may underlie IC deficits shared by MDD and PUD, whereas disorder-differentiating activation abnormalities in midcingulate and parietal regions may account for their distinct features associated with disturbed goal-directed behavior.

## Introduction

Major depressive disorder (MDD) and psychostimulant use disorder (PUD) are two distinct yet often co-occurring psychiatric conditions (Filip et al., 2014; Lin et al., 2012). MDD, characterized by depressed mood, loss of interest and pleasure, and impaired cognitive function (Otte et al., 2016), affects 6% of adults worldwide each year (Bromet et al., 2011). Psychostimulants, notably cocaine and methamphetamine, are the second most commonly used illicit substances (Patel et al., 2016). The global prevalence of psychostimulant use is reportedly 0.3–1.1% (Ashok, Mizuno, Volkow, & Howes, 2017), with PUD affecting 16% of cocaine users and 11% of amphetamine-like stimulant users (Farrell et al., 2019). Prolonged abuse of psychostimulants can lead to cognitive deficits (D’Souza, 2019) and mental health issues such as depression, anxiety, and psychosis (Ibáñez, Cáceresa, Brucher, & Seijas, 2020). Epidemiologic studies indicated that 35.7% of cocaine users have a lifetime history of depression (Conway, Compton, Stinson, & Grant, 2006; Glasner-Edwards et al., 2009), and depressive symptoms among methamphetamine users are even more severe and persistent compared to cocaine users (Kay-Lambkin et al., 2011). On the other hand, according to the ‘self-medication hypothesis’ (Markou, Kosten, & Koob, 1998), patients with MDD may turn to drugs to compensate for their anhedonia and motivational impairment (Lin et al., 2012). The co-occurrence of MDD and PUD can exacerbate the clinical symptoms and increase the risk of relapse, imposing a great burden on both individuals and society (Zilkha, Barnea-Ygael, Keidar, & Zangen, 2020).

Among the factors contributing to disability in people with depression and psychostimulant use, cognitive deficits, especially inhibitory control (IC) deficits, is a shared core feature of these disorders and considered a common risk factor (Hildebrandt, Dieterich, & Endrass, 2021; Yitzhak et al., 2023). IC is the ability to suppress inappropriate prepotent responses and resist interference of irrelevant information to enable goal-directed behaviors (Aron, 2007; Diamond, 2013), which may be associated with the failure to inhibit ruminative thoughts in MDD (Shimony et al., 2021) and higher levels of impulsivity and attentional deficits in PUD (Jovanovski, Erb, & Zakzanis, 2005; Verdejo-Garcia, Lawrence, & Clark, 2008). Cognitive models for depression highlight IC deficits as a key component in a proposed mechanism for MDD (Yitzhak et al., 2023), and a meta-analysis showed that among all cognitive domains, IC deficits had the strongest effect size in children and adolescents with MDD compared to healthy controls (HCs; Wagner et al., 2015). According to the established impaired response inhibition and salience attribution model of addiction (Goldstein & Volkow, 2002), IC deficits serve as a critical psychological mechanism in the development and persistence of substance addiction (Dai et al., 2022). Among substance use disorders, PUD shows the most common and severe IC deficits (Frazer, Richards, & Keith, 2018; Lee, Hoppenbrouwers, & Franken, 2019), while users of other substances, such as opioids, often exhibit more severe mood disturbances (Ahn & Vassileva, 2016). Numerous empirical studies have demonstrated IC alterations in both MDD and PUD (Elton et al., 2014; Liu et al., 2021a; Nestor, Ghahremani, Monterosso, & London, 2011). Meta-analytical studies provide consistent evidence that depressed patients (Dotson et al., 2020) and PUD (Smith, Mattick, Jamadar, & Iredale, 2014) performed poorly on the IC paradigms such as Stroop, Go/NoGo, and stop-signal task (SST) compared to HC, suggesting that impaired IC might be a transdiagnostic behavioral phenotype.

While both MDD and PUD involve deficient IC, their IC deficits may arise from divergent neural mechanisms. Functional magnetic resonance imaging (fMRI) studies of IC in MDD have reported altered brain activation in inferior/medial/posterior frontal, anterior cingulate, midcingulate, and inferior parietal regions during Go/NoGo and SST (Langenecker et al., 2019; Liu et al., 2021a), but with inconsistent results (Bobb et al., 2012; Piani, Maggioni, Delvecchio, & Brambilla, 2022a), for example, both greater activation (Langenecker et al., 2007; Langenecker et al., 2019) and lesser activation (Jenkins et al., 2018) in anterior cingulate cortex (ACC) during IC tasks. Two meta-analyses in MDD reveal consistent hypoactivation in ACC and anterior insula during executive control (Diener et al., 2012; Miller, Hamilton, Sacchet, & Gotlib, 2015), although they examined activation across a range of tasks and did not isolate IC. In PUD, both cocaine and methamphetamine users show hyperactivation in motor control regions such as the precentral gyrus (Brewer et al., 2008; Fassbender, Lesh, Ursu, & Salo, 2015; Morein-Zamir et al., 2015) and self-referential processing areas such as the precuneus (Morein-Zamir et al., 2015; Nestor et al., 2011), as well as hypoactivation in frontoparietal control regions including the inferior parietal lobule (IPL; Barrós-Loscertales et al., 2011; Zilverstand, Huang, Alia-Klein, & Goldstein, 2018) and dorsolateral prefrontal cortex (DLPFC; Moeller et al., 2014; Nestor et al., 2011), and conflict monitoring regions such as the ACC (Hester & Garavan, 2004; Zerekidze et al., 2023) in Stroop, Go/NoGo, or SST tasks. Furthermore, a recent meta-analysis revealed hypoactivation of the left dorsal ACC (dACC) and right middle frontal gyrus (MFG) in individuals with active drug addiction during IC tasks (Le, Potvin, Zhornitsky, & Li, 2021). An earlier review suggested that dysfunction of prefrontal regions (including dACC, DLPFC, and inferior frontal gyrus [IFG]) may contribute to impaired response inhibition in these individuals (Goldstein & Volkow, 2011).

Taken together, these studies suggest partly overlapping IC-related frontoparietal abnormalities in MDD and PUD. However, the findings are mixed, and the common and distinct neurobiological underpinnings of IC between these disorders have not been systematically determined. Given their shared phenotype and in line with the National Institute of Mental Health Research Domain Criteria framework (Kozak & Cuthbert, 2016), this study aimed to dissect shared and distinct neural substrates underlying inhibition processes within the construct of ‘cognitive control’ in a cognitive systems domain. By comparing IC-related dysfunctions in MDD and PUD, we can identify common and disorder-differentiating neuroimaging markers of IC that may potentially serve as therapeutic targets for the development of new treatments for MDD and PUD.

To this end, we conducted a quantitative meta-analysis comparing brain activation during IC tasks between MDD and PUD, using all eligible case–control whole-brain fMRI studies in both disorders. We used voxel-wise anisotropic effect size signed differential mapping (AES-SDM, https://www.sdmproject.com/software/), which allows coordinate-based meta-analyses of neuroimaging studies with unbiased estimation of effect sizes (Radua & Mataix-Cols, 2009; Radua et al., 2014). A three-step approach was employed: first, separate meta-analyses within each patient group to characterize robust activation abnormalities relative to HC; second, a quantitative comparison of activation abnormalities between MDD and PUD; and third, a conjunction/disjunction analysis of shared/contrasting abnormalities across both groups. As IC is a construct of two distinct but interconnected dimensions (Uhre et al., 2022), that is, cognitive inhibition (the suppression of competing cognitive processing) and response inhibition (the suppression of a prepotent motor response), we first examined these two dimensions together and then separately. Based on previous literature, we hypothesized that these two disorders manifest common alterations in prefrontal regions (e.g. MFG, IFG) that are engaged in cognitive control and inhibitory processes (McTeague et al., 2017; McTeague, Goodkind, & Etkin, 2016; Yan et al., 2022).

## Methods

### Literature search and selection

The meta-analyses were conducted according to the Preferred Reporting Items for Systematic Reviews and Meta-Analyses guidelines (Moher et al., 2009). A comprehensive search was conducted in PubMed, Web of Science, and Scopus databases up to March 25, 2024, for fMRI studies involving inhibition-related tasks, which compared patients with MDD or PUD with HC, respectively. The search terms are provided in the Supplementary Materials.

Studies were included if (a) a whole-brain activation comparison of patients with MDD or PUD (cocaine or methamphetamine use disorder) with HC was conducted, (b) an IC task was performed measuring cognitive inhibition (e.g. Stroop) or response inhibition (e.g. Go/NoGo, SST), (c) the *t*-map or coordinates in Talairach or Montreal Neurological Institute (MNI) space were provided, and (d) the study was published in a peer-reviewed English-language journal. Studies were excluded if (a) they were nonempirical studies (e.g. review, meta-analysis) or preliminary studies (e.g. meeting abstract), (b) they were nonhuman studies, (c) the sample repeated that in a previous study, (d) only the region-of-interest approach was used, and (e) there was no IC contrast.

### Data selection and extraction

Two authors (Y.L. and X.L.) independently screened and assessed all search results, achieving 100% agreement. From the selected articles, these authors independently extracted peak coordinates of significant activation differences and effect sizes; demographic and clinical information (e.g. sample size, mean age, comorbidity, medication, age of onset, and duration of illness); and scanning technicalities (e.g. scanner field strength, smoothing, and task).

### Meta-analyses

The voxel-wise meta-analyses were conducted using AES-SDM v.5.15 (https://www.sdmproject.com/software/). AES-SDM is a powerful statistical technique to synthesize diverse neuroimaging findings (Radua et al., 2012) and has been widely used in previous meta-analyses (Liloia et al., 2024; Liu et al., 2021b; Pan et al., 2024; Schrammen et al., 2022). It utilizes reported peak coordinates and *t*-values from included studies to recreate mean effect-size maps using an anisotropic Gaussian kernel; the mean maps are then weighted by sample size, intrastudy variance, and interstudy heterogeneity and compared to a null distribution with a random-effect model (Radua et al., 2012; Radua et al., 2014).

We employed a three-step meta-analytic approach to determine distinct and shared brain activation alterations during IC tasks in patients with MDD and PUD. First, to assess robust brain activation alterations for each patient group relative to their HC, separate meta-analyses (MDD vs. HC and PUD vs. HC) were conducted using results from IC-related contrasts (e.g. NoGo vs. Go, incongruent vs. congruent, and stop vs. failed-stop). Second, to investigate differentiating activation alterations, a quantitative comparative meta-analysis comparing brain activation between patients with MDD and PUD was conducted, while covarying for age and gender. Third, to identify overlapping/contrasting neurofunctional alterations between patients with MDD and PUD, a conjunction/disjunction analysis was performed, taking into account the error in estimating the *p*-values from individual meta-analyses. Prior to meta-analyses, Talairach coordinates were converted to MNI space, and other effect-size measures (e.g. *z-*values) were converted to *t*-values using the online calculator provided by AES-SDM. In the separate-group meta-analyses and between-group comparative analyses, we applied default parameters and thresholds (full-width at half-maximum = 20 mm, voxel-wise *p* < 0.005, SDM-Z > 1, and a cluster extent size of ≥10 voxels (Radua et al., 2012; Radua et al., 2014). In the conjunction/disjunction analysis, in line with previous studies (Bore et al., 2023; Liu et al., 2022), we used a more stringent threshold of *p* < 0.0025 and a cluster extent size of ≥10 voxels.

### Sensitivity analyses

To examine the potential confounding effects of demographics and clinical variables on brain activation alterations, we conducted meta-regression analyses within each patient group, with regressor variables that were reported in at least nine studies (Radua & Mataix-Cols, 2009): mean age, gender ratio, and comorbidity ratio for both groups; and abstinence days and duration of psychostimulant use additionally for PUD. The threshold was set at voxel-wise *p* < 0.0005 and a cluster extent size of ≥20 voxels, and only regions found in the main meta-analyses were included (Radua et al., 2012). Several subgroup analyses were performed to control for potential confounding effects of medication, psychostimulant type (cocaine vs. methamphetamine) and dimension of inhibition (response inhibition vs. cognitive inhibition). Considering that comorbidity may influence both common and distinct brain activation patterns, we also controlled for the comorbidity ratio in the comparative and conjunctive meta-analyses (for detailed descriptions and results, see Supplementary Methods and Table S3).

We used a leave-one-out jackknife sensitivity analysis to evaluate the impact of each study on the overall results. We used a random-effect model with Q statistics to quantify interstudy heterogeneity. The heterogeneous brain regions were identified using the default threshold parameters (full-width at half-maximum = 20 mm, voxel-wise *p* < 0.005, peak height SDM-Z > 1, and a cluster extent size of ≥10 voxels). Funnel plots and Egger’s test were used to assess potential publication bias, setting significance at *p* < 0.05.

## Results

### Included studies and sample characteristics

**Figure 1.**
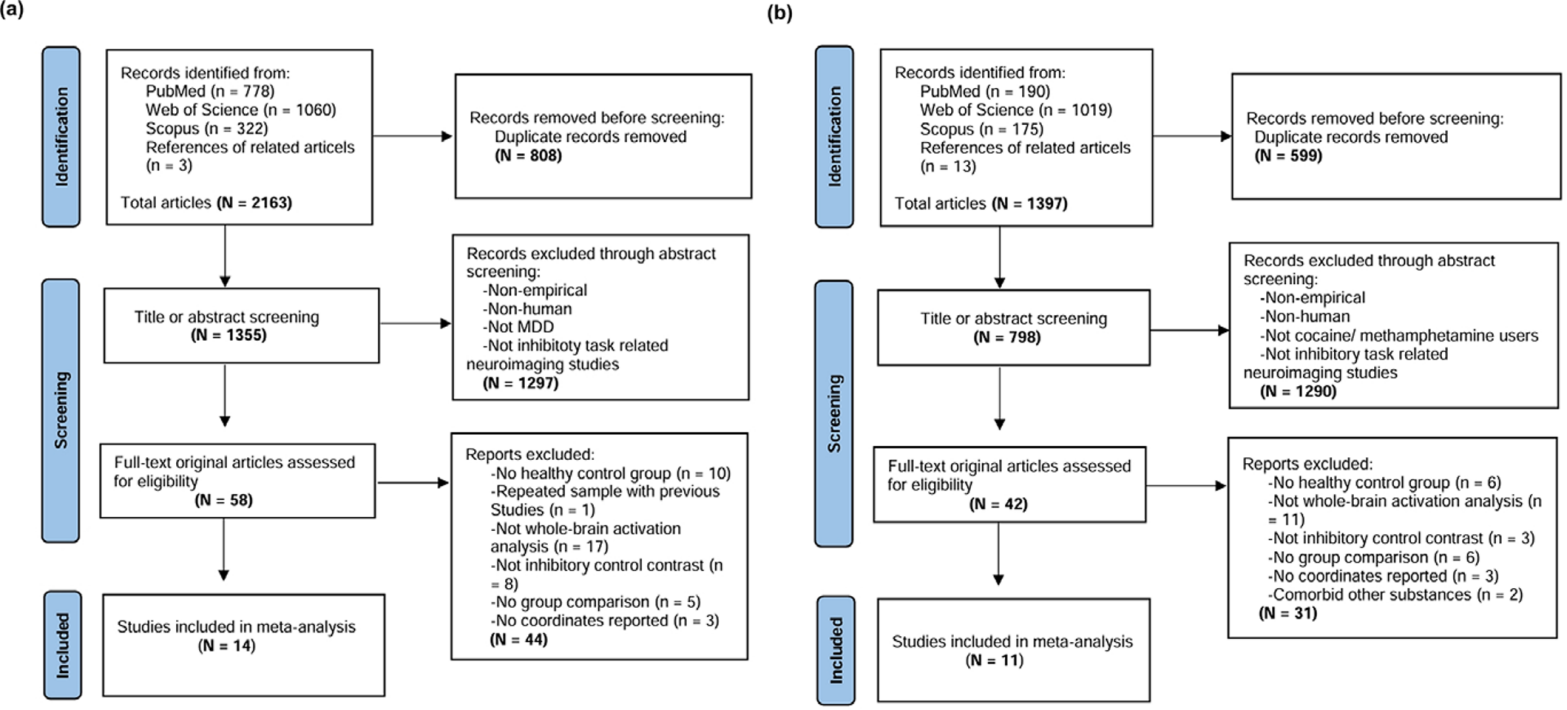
PRISMA flowchart. Literature searching and selection process for (a) major depressive disorder (MDD) and (b) psychostimulant use disorder (PUD).

A literature search yielded 3560 studies (2163 MDD and 1397 PUD), of which 14 MDD and 11 PUD studies met all the inclusion criteria for meta-analysis (Figure 1).

Tables 1 and 2 summarize demographic and clinical information for participants. The MDD studies (Table 1) comprised 17 datasets comparing activation in IC tasks between 340 patients with MDD (228 females, mean age 30.8 years) and 303 HCs (192 females, mean age 28.4 years). The PUD studies (Table 2) comprised 12 datasets comparing 258 patients with PUD (71 females, mean age 37.7 years) and 273 HCs (88 females, mean age 35.8 years). Among the PUD studies, three studies (including three datasets) focused on methamphetamine users, and the remaining eight studies (including nine datasets) investigated cocaine users.

**Table 1.** Demographic and clinical characteristics of the 14 task-fMRI MDD studies included in the meta-analysis (17 datasets, 340 patients with MDD)

Study	MDD							HC		Scanner/FWHM (mm)	Task	Threshold	Summary findings
	Number (female)	Mean (SD) age (y)	Medication (Yes/No)	Diagnosis (type)	Comorbidity	Mean (SD) age at onset (y)	Mean (SD) duration (y)	Number (female)	Mean (SD) age (y)				
Bobb et al. (2012)	15 (12)	60.7 (4.7)	No	Research criteria (MDD)	No	NA	NA	13 (9)	62.0 (5.3)	3 T/6	SST	*p* < 0.01 (corr)	HC < MDD: L ACC, L MFG, L MOFG, L Cd, L MFG
Cha et al. (2021)	28 (24)	23.1 (3.5)	No	NA	Twenty-eight STM	14.7 (4.3)	8.4 (5.6)	29 (18)	24.8 (3.4)	3 T/8	Go/NoGo	*p* < 0.05 (Bonferroni)	-
	41 (27)	25.1 (3.8)			NA	15.3 (4.4)	9.8 (5.4)						HC < MDD: R TP
Chechko et al. (2013)	18 (13)	36.5 (10.8)	Yes (n = 12)	DSM-IV (MDD)	No	30.3 (7.5)	NA	18 (13)	36.0 (10.3)	3 T/8	Stroop	*p* < 0.05 (FWE)	HC > MDD: R CB, L CB, L IFG, R IFG MFG, L IPL, R IPL, L SMA, R ITG
Crane et al. (2016)	29 (21)	33.2 (11.3)	No	DSM-IV (MDD)	Twelve anxiety, five SOP, four GAD, four NA, two PD, two SIP	NA	NA	54 (38)	33.8 (11.6)	3 T/5	Go/NoGo	*p* < 0.005 (Alphasim)	HC > MDD: R PCC
	18 (11)	34.3 (11.7)			NA								HC > MDD: R SFG, R MFG, R PCC, R Cuneus, L FG, L Cd
Diler et al. (2014)	10 (8)	15.9 (1.1)	Yes (n = 6)	DSM-IV (MDE)	Six Anxiety, two ADHD	NA	0.27 (0.12)^a^	10 (8)	15.6 (1.1)	3 T/6	Go/NoGo	*p* < 0.008 (corr)	HC < MDD: L STG, L Cd, L Occipital
Halari et al. (2009)	21 (11)	16.2 (0.8)	No	DSM-IV (MDD)	No	NA	0	21 (11)	16.3 (1.1)	1.5 T/7.2	SST	*p* < 0.05 (NA)	HC > MDD: R DLPFC, R SPL, ACC/MFL
Langenecker et al. (2007)	22 (15)	41.0 (12.2)	No	DSM-IV (MDD)	NA	27.9 (15.1)	13 (NA)	22 (14)	34.2 (11.0)	3 T/NA	Go/NoGo	*p* < 0.0001 (uncorr)	HC < MDD: L ACC, R IFG STG, L Postcentral, R Insula, R MTG, L FG
Langenecker et al. (2018)	21 (14)	21.0 (1.4)	Yes (n = 9)	NA	NA	16.3 (3.7)	NA	39 (23)	14.83 (1.25)	3 T/NA	Go/NoGo	*p* < 0.0001 (NA)	HC < MDD: L SGAC
Malejko et al. (2022)	13 (10)	16.2 (1.4)	Yes (n = 10)	DSM-V (MDD)	Five minor NSSI, two anxiety, two ED, one ADHD, four PTSD	NA	NA	14 (11)	14.4 (1.8)	3 T/8	Go/NoGo	*p* < 0.05 (uncorr)	HC < MDD: L AIC, L Postcentral, L Precuneus, L IPC, L CB
Matthews et al. (2009)	15 (12)	24.5 (NA)	No	DSM-IV (MDD)	Three dysthymia, one PD and GAD, two PTSD, one PD and dysthymia	NA	NA	16 (10)	24.3 (NA)	3 T/6	SST	*p* < 0.05 (NA)	HC < MDD: SGAC
Pan et al. (2011)	15 (8)	15.87 (1.55)	Yes (n = 7)	DSM-IV (MDD)	No	NA	NA	14 (6)	15.21 (1.42)	3 T/6	Go/NoGo	*p* < 0.05 (corr)	HC < MDD: L ACC, R ACC, L Insula
Piani et al. (2022b)	12 (5)	66.5 (9.1)	Yes (n = 11)	DSM-V (MDD)	No	NA	NA	12 (7)	68.7 (12.3)	3 T/4	Go/NoGo	*p* < 0.001 (NA)	HC < MDD: R LG
Richard-Devantoy et al. (2015)	26 (15)	40.3 (9.7)	No	DSM-IV (MDE)	No	30.6 (13.2)	NA	28 (17)	33.8 (7.1)	3 T/8	Go/NoGo	*p* < 0.05 (FWE)	HC > MDD: precuneus MCC/PCC. HC < MDD: R IFG MPFC& bilateral MFG, L IFG, bilateral SMA AG PCC, precuneus
	23 (15)	41.3 (11.4)				37.9 (10.1)							HC > MDD: SMA, MCT; HC < MDD: L IPL, R IPL
Yang et al. (2009)	13 (7)	16.0 (1.5)	No	DSM-IV (MDD)	No	NA	NA	13 (7)	15.8 (1.5)	3 T/4	SST	*p* < 0.05 (corr)	HC > MDD: MFG, L VC; HC < MDD: L ACC

Abbreviations: ACC, ‘anterior cingulate cortex’; ADHD, ‘attention deficit and hyperactivity disorder’; AG, ‘angular gyrus’; AIC, ‘anterior insula cortex’; CB, ‘cerebellum’; Cd, ‘caudate’; corr, ‘corrected’; DLPFC, ‘dorsolateral prefrontal cortex’; ED, ‘eating disorders’; FG, ‘fusiform gyrus’; DSM, ‘Diagnostic and Statistical Manual of Mental Disorders’; FWHM, ‘full width at half maximum’; FWE, ‘family-wise error’; GAD, ‘generalized anxiety disorder’; HC, ‘healthy control’; IFG, ‘inferior frontal gyrus’; IPC, ‘inferior parietal cortex’; IPL, ‘inferior parietal lobule’; ITG, ‘inferior temporal gyrus’; L, ‘left’; LG, ‘lingual gyrus’; MCC, ‘middle cingulate cortex’; MCT, ‘midline caudate thalamus’; MDD, ‘major depressive disorder’; MDE, ‘major depressive episode’; MFG, ‘middle frontal gyrus’; MFL, ‘mesial frontal lobe’; MOFG, ‘middle orbitofrontal gyrus’; MPFC, ‘medial prefrontal cortex’; MTG, ‘middle temporal gyrus’; NA, ‘not available’; NSSI, ‘non-suicidal self-injury’; PCC, ‘posterior cingulate cortex’; PD, ‘panic disorder’; PTSD, ‘post-traumatic stress disorder’; R, ‘right’; SD, ‘standard deviation’; SGAC, ‘subgenual anterior cingulate’; SFG, ‘superior frontal gyrus’; SIP, ‘simple phobia’; SMA, ‘supplementary motor area’; SOP, ‘social phobia’; SPL, ‘superior parietal lobe’; SST, ‘stop-signal task’; STG, ‘superior temporal gyrus’; STM, ‘subthreshold mania’; TP, ‘temporal pole’; uncorr, ‘uncorrected’; VC, ‘visual cortex’; y, ‘years’.

a Indicates current illness episodes.

**Table 2.** Demographic and clinical characteristics of the 11 task-fMRI PUD studies included in the meta-analysis (12 datasets, 258 PUD)

Study	PUD								HC		Scanner/FWHM (mm)	Task	Threshold	Summary findings
	Number (female)	Mean (SD) age (y)	Substance	Diagnosis (type)	Comorbidity	Length of abstinence	Mean (SD) age at onset (y)	Years (SD) of use (y)	Number (female)	Mean (SD) age (y)				
Barros-Loscertales et al. (2011)	16 (0)	34.4 (7.2)	Cocaine	DSM-IV (NA)	No	> 2–4 d	19.9 (6.3)	13.9 (5.9)	16 (0)	34.2 (8.9)	1.5 T/9	Stroop	*p* < 0.005 (corr)	HC > PUD: R IFG, R IPG, R STG
Ceceli et al. (2022)	26 (4)	44.1 (8.2)	Cocaine	DSM-IV (SUD)	Five IED, two specific phobias, one marijuana dependence	Mean 293 d (42% users)	NA	16.0 (8.2)	26 (5)	42.7 (7.1)	3 T/5	SST	*p* < 0.005 (corr)	HC > PUD: L LOC, L FP/DMPFC
Connolly et al. (2012)	9 (2)	36.4 (6.6)	Cocaine (short-term abstinent)	NA	NA	Mean 2.4 w	NA	12.1 (5.0)	9 (2)	30.5 (6.7)	1.5 T/4.2	Go/NoGo	*p* < 0.05 (corr)	HC < PUD: R MFG, R SFG, R precentral, R MTG
	9 (2)	32.8 (8.3)	Cocaine (long-term abstinent)	NA		Mean 69 w	NA	10.6 (7.6)						HC > PUD: L STG; HC < PUD: R IFG, R MFG, R Precentral, bilateral cerebellar tonsil
Fassbender et al. (2015)	30 (15)	35.5 (7.9)	MA	DSM-IV (MA-D)	No	Mean 14 m	17.7 (4.4)	14.0 (6.4)	27 (11)	28.5 (7.2)	3 T/8	Stroop	*p* < 0.05 (FWE)	HC < PUD: L precentral, PCC, L MTG/ITG
Ide et al. (2016)	75 (25)	39.9 (7.6)	Cocaine	DSM-IV (C-D)	No	Mean 18 d	NA	18.0 (8.2)	88 (39)	38.7 (10.9)	3 T/6	SST	*p* < 0.05 (FWE)	HC > PUD: R AG, R IFG, R SMG, L MOC, L SMG, L MOC, L IFG, L IFC, R LOC, R LFC
Jan et al. (2014)	15 (4)	35.3 (7.0)	MA	DSM-IV (MA-D)	No	Current	23.3 (7.2)	10.8 (5.8)	18 (6)	31.1 (8.1)	1.5 T/8	Stroop	*p* < 0.05 (corr)	HC < PUD: R SFG, R MFG
Li et al. (2007)	15 (0)	37.7 (6.8)	Cocaine	DSM-IV (C-D)	No	> 2 w	NA	10.2 (7.3)	15 (0)	36.6 (6.0)	3 T/10	SST	*p* < 0.001 (uncorr)	HC > PUD: L AG, L SMG, L PACG, L LG, RSMG
Ma et al. (2015)	13 (1)	37.4 (5.3)	Cocaine	DSM-IV (C-D)	No	Current	NA	NA	10 (3)	35.2 (7.3)	3 T/8	Go/NoGo	*p* < 0.05 (FWE)	HC > PUD: R precentral, R MFG, R MCC, R SFG, R paracentral lobe
Morein-Zamir et al. (2015)	24 (12)	28.5 (6.8)	Cocaine	NA	No	Current	20.42 (3.39)	8.1 (6.2)	31 (14)	30.9 (8.1)	3 T/8	SST	*p* < 0.001 (uncorr)	HC < PUD: R ACC, L FG R pre-SMA, L precentral R MCC, R SFG, L SFG, R hippocampus, R SMG, L SMG, R Occipital, R IOG
Moeller et al. (2012)	16 (1)	46.3 (7.8)	Cocaine	DSM-IV (C-D)	One heroin dependence	< 25 d	27.4 (7.2)	15.6 (8.3)	15 (1)	38.9 (7.1)	4 T/8	Stroop	*p* < 0.05 (FDR)	HC > PUD: R IFG, R SMG, R precentral, SMA/ACC, R FOC, R IC R OP, R LOC, R IFG, R TCFC, R MTG, R TP,
Nestor et al. (2011)	10 (5)	33.5 (9.3)	MA	DSM-IV (MA-D)	No	4–7 d	NA	8.3 (3.7)	18 (7)	36.4 (10.4)	3 T/6	Stroop	*p* < 0.05 (corr)	HC < PUD: L putamen, R cerebellum, bilateral insula, R LG, L CF, L FG, R cuneus, R SOG, bilateral DLPFC, L LG, L IOC, SMFG, R precuneus

Abbreviations: ACC, ‘anterior cingulate cortex’; AG, ‘angular gyrus’; C-D, ‘cocaine dependence’; CF, ‘calcarine fissure’; corr, ‘corrected’; DLPFC, ‘dorsolateral prefrontal cortex’; DMPFC, ‘dorsal medial prefrontal cortex’; d, ‘days’; DSM, ‘Diagnostic and Statistical Manual of Mental Disorders’; FG, ‘fusiform gyrus’; FOC, ‘frontal operculum cortex’; FP, ‘frontal pole’; FWE, ‘family-wise error’; FWHM, ‘full width at half maximum’; HC, ‘healthy control’; IC, ‘insula cortex’; IED, ‘intermittent explosive disorder’; IFC, ‘inferior frontal cortex’; IFG, ‘inferior frontal gyrus’; IOC, ‘inferior occipital cortex’; IOG, ‘inferior occipital gyrus’; IPG, ‘inferior parietal gyrus’; ITG, ‘inferior temporal gyrus’; LFC, ‘lateral frontal cortex’; LG, ‘lingual gyrus’; LOC, ‘lateral occipital cortex’; MA, ‘methamphetamine’; MA-D, ‘methamphetamine dependence’; MCC, ‘middle cingulate cortex’; MFG, ‘middle frontal gyrus’; MOC, ‘middle occipital cortex’; m, ‘months’; MTG, ‘middle temporal gyrus’; NA, ‘not available’; OP, ‘occipital pole’; PACG, ‘perigenual anterior cingulate gyrus’; PTSD, ‘post-traumatic stress disorder’; PUD, ‘psychostimulant use disorder’; SD, ‘standard deviation’; SFG, ‘superior frontal gyrus’; SMG, ‘supramarginal gyrus’; SMA, ‘supplementary motor area’; SMFG, ‘superior medial frontal gyrus’; SOG, ‘superior occipital gyrus’; SST, ‘stop-signal task’; STG, ‘superior temporal gyrus’; SUD, ‘substance use disorder’; TCFC, ‘temporal occipital fusiform cortex’; THC, ‘delta-9-tetrahydrocannabinol’; TP, ‘temporal pole’; uncorr, ‘uncorrected’; w, ‘weeks’.

There were no significant differences in mean age or gender ratio between patients with MDD and HC (mean age: *t* = 0.030, *p* = 0.766; gender ratio: *t* = 1.111, *p* = 0.276) or between patients with PUD and HC (mean age: *t* = 1.007, *p* = 0.326; gender ratio: *t* = −0.810, *p* = 0.936) using a weighted two-sample *t*-test. Between the two patient groups, there was no significant difference in mean age (*t* = −1.440, *p* = 0.165); however, MDD studies had a higher proportion of females than that in PUD studies (*t* = 5.706, *p* < 0.001).

### Brain activation alterations

#### Comparing MDD versus HC

**Figure 2.**
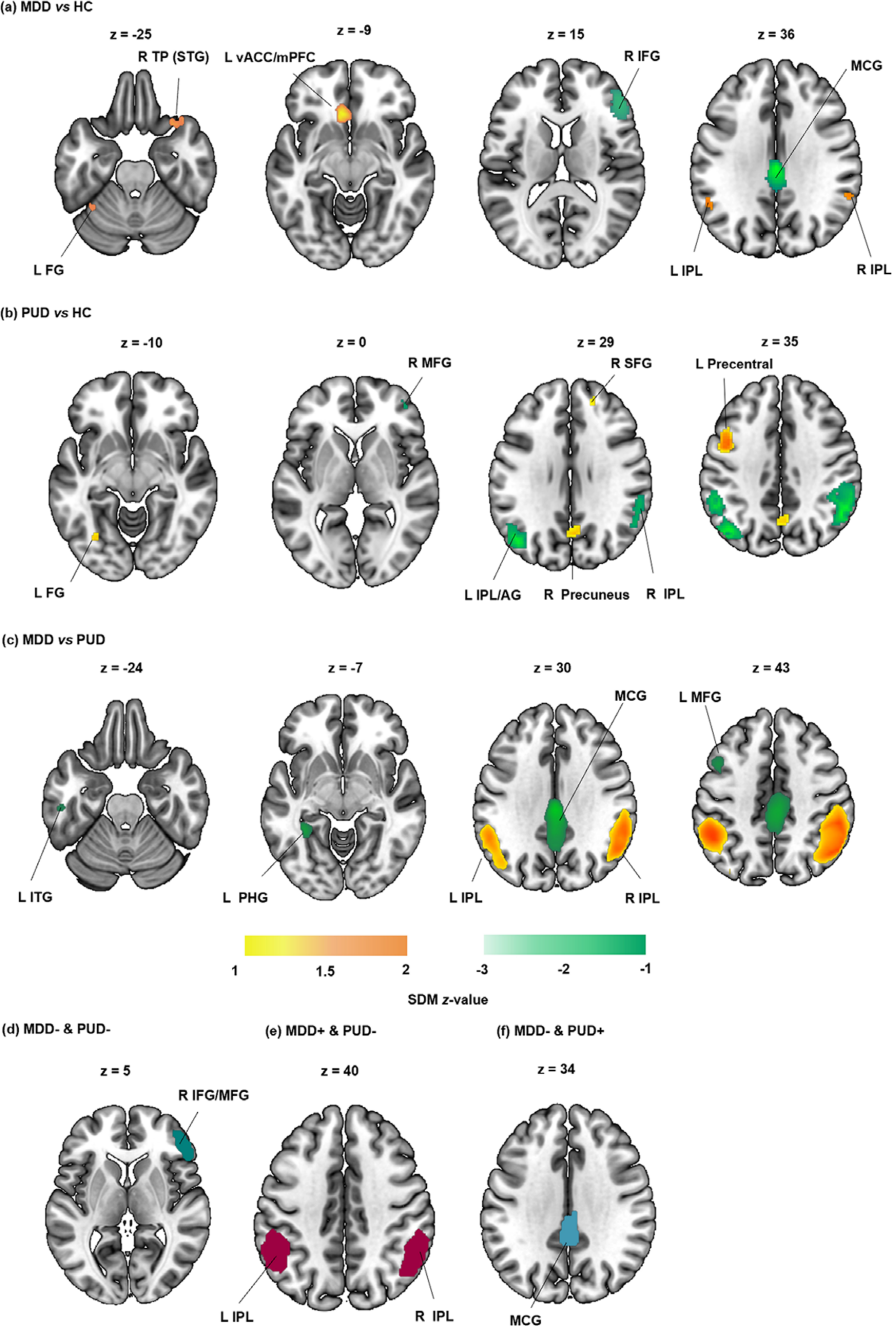
Shared and distinct brain activation alterations between groups during inhibitory control tasks. The first three panels show brain activation alterations (a) in major depressive disorder (MDD) relative to healthy controls (HCs), (b) in psychostimulant use disorder (PUD) relative to HC, and (c) between MDD (vs. HC) and PUD (vs. HC) covarying for mean age and gender ratio. The heat scale reflects the (positive and negative) SDM z-value. The last three panels show conjunction/disjunction in changes (vs. HC): (d) hypoactivation in both groups, (e) hyperactivation in MDD and hypoactivation in PUD, and (f) hypoactivation in MDD and hyperactivation in PUD. Separate-group meta-analyses and between-group comparative analyses are shown at *p* < 0.005, and conjunctive meta-analyses at *p* < 0.0025. Other abbreviations: AG, ‘angular gyrus’; FG, ‘fusiform gyrus’; IFG, ‘inferior frontal gyrus’; IPL, ‘inferior parietal lobule’; ITG, ‘inferior temporal gyrus’; L, ‘left’; MCG, ‘median cingulate/paracingulate gyri’; MFG, ‘middle frontal gyrus’; mPFC, ‘medial prefrontal cortex’; PCC, ‘posterior cingulate cortex’; PHG, ‘parahippocampal gyrus’; R, ‘right’; SFG, ‘superior frontal gyrus’; STG, ‘superior temporal gyrus’; TP, ‘temporal pole’; vACC, ‘ventral anterior cingulate cortex’.

Relative to HC, MDD showed hyperactivation in the left ventral anterior cingulate cortex/medial prefrontal cortex (vACC/mPFC), bilateral IPL, right temporal pole/superior temporal gyrus (STG) and left fusiform gyrus (FG), and hypoactivation in median cingulate/paracingulate gyri (MCG) and right IFG (Figure 2a; Table 3).

**Table 3.** Whole-brain meta-analysis results for task-fMRI studies in MDD and PUD

MNI coordinates	SDM-Z	Voxels	Regions (voxel threshold p < 0.005, cluster size ≥ 10 voxels)	BA	Egger’s bias	Egger’s p	Jackknife sensitivity
Comparison of MDD vs. HC							
**MDD > HC**							
−6, 28, −8	1.432	196	L ventral anterior cingulate cortex/medial prefrontal cortex	11	−0.00	~1	15/17
−48, −50, 42	1.336	197	L inferior parietal lobule	40	−0.37	0.71	15/17
40, 22, −26	1.200	70	R temporal pole/superior temporal gyrus	38	−1.23	0.38	16/17
58, −46, 30	1.177	59	R inferior parietal lobule	48	−0.19	0.89	14/17
−38, −52, −24	1.159	18	L fusiform gyrus	37	1.75	0.16	16/17
**MDD < HC**							
0, −24, 32	−1.865	1320	Median cingulate/paracingulate gyri	\	1.35	0.06	17/17
50, 34, 14	−1.384	663	R inferior frontal gyrus, triangular part	45	−0.99	0.41	15/17
**Comparison of PUD vs. HC**							
**PUD > HC**							
−48, 12, 46	1.750	470	L precentral gyrus	9	1.08	0.27	11/12
6, −64, 34	1.243	195	R precuneus	7	0.47	0.54	11/12
−32, −68, −6	1.222	34	L fusiform gyrus	\	0.18	0.83	10/12
18, 46, 28	1.201	15	R superior frontal gyrus, dorsolateral	\	1.19	0.25	9/12
**PUD < HC**							
52, −54, 44	−2.998	1577	R inferior parietal lobule	40	0.76	0.30	12/12
−46, −70, 36	−2.927	1168	L inferior parietal lobule/angular gyrus	39	1.50	0.01	12/12
42, 48, 0	−1.967	30	R middle frontal gyrus	46	1.79	~0	12/12
36, 58, 10	−1.887	18	R middle frontal gyrus	10	1.97	~0	11/12
**Comparison of MDD vs. PUD**							
**MDD > PUD**							
52, −58, 46	2.611	2477	R inferior parietal lobule	40	0.38	0.55	
−50, −50, 42	2.622	1493	L inferior parietal lobule	40	0.98	0.05	
−36, −78, 46	1.391	10	L inferior parietal lobule	\	2.22	~0	
**MDD < PUD**							
0, −26, 32	−2.081	1707	Median cingulate/paracingulate gyri	\	0.30	0.45	
−34, −46, −4	−1.519	113	L parahippocampal gyrus	\	0.02	0.96	
−46, 12, 44	−1.346	107	L middle frontal gyrus	9	0.51	0.37	
−54, −24, −24	−1.087	19	L inferior temporal gyrus	20	0.00	~1	

Abbreviations: BA, ‘Brodmann areas’; HC, ‘healthy control’; L, ‘left’; PUD, ‘psychostimulant use disorder’; SDM, ‘seed-based d mapping’; R, ‘right’.

#### Comparing PUD versus HC

Relative to HC, PUD showed hyperactivation in the left precentral gyrus, right precuneus, left FG and right superior frontal gyrus (SFG), and hypoactivation in the right IPL, left IPL/angular gyrus (AG), and right MFG (Figure 2b; Table 3).

#### Comparing MDD versus PUD

Covarying for mean age and gender ratio, MDD showed hyperactivation relative to PUD in the bilateral IPL, and hypoactivation relative to PUD in the MCG, left parahippocampal gyrus (PHG), left MFG, and left inferior temporal gyrus (ITG) (Figure 2c; Table 3). Moreover, the differential results between MDD and PUD remained robust even after additionally controlling for the comorbidity ratio (together with mean age and gender ratio) in comparative meta-analyses (Supplementary Table S3).

#### Conjunction/disjunction analyses

In conjunction analysis, there was convergent hypoactivation in the right IFG/MFG (MNI: 46, 44, 6; voxels: 1002; BA45; Figure 2d) in both patient groups. In disjunction analysis, MDD showed hyperactivation, while PUD showed hypoactivation in the right IPL (MNI: 60, −44, 34; voxels: 1653; BA40; Figure 2e) and left IPL (MNI: −48, −50, 42; voxels: 1423; BA40; Figure 2e). In the reverse pattern, PUD showed hyperactivation and MDD showed hypoactivation in the MCG (MNI: 0, −42, 32; voxels: 591; BA23; Figure 2f). Excluding studies with comorbid patients confirmed the highly robust patterns (Supplementary Table S3).

### Mapping results onto large-scale networks

To provide a functional interpretation of the results, we mapped the identified brain regions onto the seven large-scale brain cortical functional networks in the Yeo 7-network parcellation atlas (Yeo et al., 2011): visual network (VN), somatomotor network (SMN), dorsal attention network (DAN), salience network (SAN), limbic network (LN), frontoparietal network (FPN), and default mode network (DMN). As identified clusters often span several networks, we calculated the percentage of voxels in each cluster that fall into each of the seven networks (results are provided in Supplementary Table S1).

### Sensitivity analyses

Meta-regression analysis was performed to identify brain regions where activation abnormalities were modulated by demographic and clinical variables in MDD and PUD, respectively (Supplementary Table S2). For MDD, a higher proportion of women was associated with greater activation in the right MFG, and there was no significant effect of mean age and comorbidity ratio. For PUD, older patients showed less activation in the right SFG; a higher comorbidity ratio was associated with greater activation in the right IPL; longer duration of psychostimulant use was primarily associated with less activation in the left IPL; longer abstinence was mainly associated with increased activation in the left MFG; and there was no significant effect of the gender ratio. Subgroup meta-analyses showed no consistent evidence for the influence of medication in MDD, whereas the PUD results were mainly driven by studies on cocaine users and response inhibition tasks, possibly due to their larger numbers (Supplementary Results and Tables S4–S6). We further controlled for comorbidity effects in the comparative and conjunctive meta-analyses, which yielded robust results as reported above (see also Supplementary Table S3). According to jackknife sensitivity analyses, the main results were highly replicable (>14 combinations for MDD and >9 combinations for PUD; Table 3). In heterogeneity analysis (Supplementary Table S7), MDD studies showed significant heterogeneity in the left cerebellum and right IPL; PUD studies showed significant heterogeneity mainly in the left SFG, right postcentral gyrus, and precuneus. In Egger’s test (Table 3), PUD studies showed significant publication bias in the left IPL/AG (*p* = 0.012), right MFG (*p* = 0.002), and right SFG (*p* = 0.001); in MDD studies, there was no evidence of publication bias (*p* > 0.05).

## Discussion

To our knowledge, this is the first neuroimaging meta-analysis to define shared and distinct neurofunctional abnormalities of IC in MDD and PUD. During IC tasks, MDD and PUD shared hypoactivation relative to HC in the right IFG/MFG, while MDD-differentiating hypoactivation was found in MCG relative to both HC and PUD, and PUD-differentiating hypoactivation was found in bilateral IPL relative to both HC and MDD. PUD also showed hyperactivation in the left PHG, MFG, and ITG relative to MDD. These results provide novel insights into the common and distinct neural basis of IC in MDD and PUD, which may facilitate a mechanistic understanding of the two disorders.

### Common brain activation abnormalities in MDD and PUD

The overlapping hypoactivation in the right IFG/MFG in MDD and PUD during IC tasks supports our hypothesis and aligns with several previous studies. Transdiagnostic neuroimaging meta-analyses have reported hypoactivation in the right IFG/MFG during cognitive control or IC tasks across multiple nonpsychotic disorders (including MDD, substance use disorders, bipolar disorder, and anxiety disorders) (McTeague et al., 2017; Yan et al., 2022). The hypoactivation patterns of right IFG/MFG during IC tasks have also been widely reported in other individual studies on MDD (Kikuchi et al., 2012) and PUD (Elton et al., 2014; Zerekidze et al., 2023). Evidence from structural MRI studies has suggested the right IFG recruitment in executive function in MDD (Vasic, Walter, Höse, & Wolf, 2008) and motor IC in PUD (Tabibnia et al., 2011). The right IFG is part of the frontoparietal cognitive control network (see Supplementary Table S1 for large-scale network mapping results), which is thought to be directly involved in inhibition and attentional control (Hampshire et al., 2010), whereas the right MFG – corresponding to the right DLPFC – supports goal maintenance and top-down cognitive control (Cole & Schneider, 2007). In a broader range of nonpsychotic disorders, the IFG/MFG serves as a critical node in the multiple-demand cognitive control/processing network, which functions as a ‘common core’ recruited across diverse cognitive challenges (McTeague et al., 2016; McTeague et al., 2017). Dysfunction in the IFG/MFG may disrupt the coordination with other networks, thereby impairing information processing and response selection in goal-directed behavior (McTeague et al., 2016; McTeague et al., 2017). Notably, the overlapping results remain highly stable when studies with comorbid samples were excluded in the conjunction meta-analyses, arguing against the comorbidity effects on the common activation alterations during IC. Together, the convergent hypoactivation pattern in the right IFG/MFG extends previous findings and suggests this region as a transdiagnostic marker of IC in MDD and PUD.

### Distinct brain activation abnormalities in MDD versus PUD

The finding of MDD-differentiating hypoactivation in MCG relative to PUD and HC adds to previous literature demonstrating reduced MCG activation during Go/NoGo in the negative context in MDD compared with generalized anxiety disorder and HC (Li, Chen, & Yan, 2022; Liu et al., 2021a). The MCG serves as a convergence hub for multiple important networks, including FPN, DMN, and SAN (Shackman et al., 2011), as supported by our large-scale network analysis (Supplementary Table S1). It has been involved in brain resource allocation (Touroutoglou, Andreano, Dickerson, & Barrett, 2020), particularly in executive function (Fedeli et al., 2022) and negative emotion regulation (Chen et al., 2007; Pereira et al., 2010). Previous meta-analytic evidence suggests that MCG is activated during a range of response-inhibition tasks in healthy populations (Zhang, Geng, & Lee, 2017), while a transdiagnostic meta-analysis demonstrated abnormal MCG activation in cognitive control across psychiatric disorders, including MDD (McTeague et al., 2017). The present comparative meta-analysis extends previous studies by showing that the IC-related MCG activation was lower in MDD (vs. HC) compared with PUD (vs. HC), and the disjunctive analysis further revealed increased MCG activation in PUD relative to HC, which may be related to different pathophysiological mechanisms in MDD and PUD despite their shared IC deficits. MDD is characterized by a rumination-related persistent state of negative mood (Marx et al., 2023), which has been found to be associated with reduced gray matter volume in MCG (Kühn, Vanderhasselt, De Raedt, & Gallinat, 2012; Liu et al., 2022). Given that the MCG has been a pivotal node of interaction between negative emotion and motor signals (Pereira et al., 2010) and negative emotion and IC competes for limited MCG resources (Tolomeo et al., 2016), the MCG-differentiating hypoactivation during IC tasks in MDD may reflect a failure to reallocate attention and effort from internal, ruminative processes to external, goal-directed cognitive demands. Conversely, increased MCG activation in PUD may reflect a compensatory overengagement of salience and control networks to suppress impulsive tendencies (Roberts & Caravan, 2010), a key feature of PUD (Verdejo-Garcia et al., 2008).

In contrast, the disorder-differentiating hypoactivation in bilateral IPL during IC in PUD relative to patients with MDD may be related to motor impulsivity and more pronounced control-related attentional deficits in PUD (Barrós-Loscertales et al., 2011; Bell, Garavan, & Foxe, 2014). This finding extends a previous systematic review reporting decreased recruitment of IPL during tasks in people with drug addiction (Zilverstand et al., 2018). The disorder-differentiating IPL, especially the right IPL, is mainly located within the FPN and DAN (Singh-Curry & Husain, 2009), which accords with our large-scale network analysis (Supplementary Table S1). The right IPL has been implicated in processes of stimulus-driven attentional reorienting (Numssen, Bzdok, & Hartwigsen, 2021), detecting salient or novel events (Singh-Curry & Husain, 2009), and response inhibition and switching (Swick, Ashley, & Turken, 2011). Previous large-scale neuroimaging meta-analyses have revealed converging activity of the IPL in different inhibition components in healthy subjects (Hung, Gaillard, Yarmak, & Arsalidou, 2018), whereas altered IPL activity during IC and error processing was reported in people with substance dependence and behavioral addictions (Luijten et al., 2014). The PUD-differentiating attenuated IPL activation observed in this study may thus explain their impairments in shifting attention away from automatic or impulsive responses due to the neurocognitive effects of chronic stimulant use.

Notably, the disorder-differentiating regions were only present in response inhibition tasks, but not in cognitive inhibition tasks, according to subgroup meta-analyses. Response/motor inhibition has been suggested to be an observable aspect of a general higher-order neurocognitive factor associated with goal maintenance (Friedman & Miyake, 2017; Miyake & Friedman, 2012). Together, the disorder-differentiating circuits of IC (particularly response inhibition) in MDD and PUD highlight distinct pathways that may lead to disrupted goal maintenance, which affects IC performance. However, these findings need to be interpreted with caution as differences in task paradigms and clinical conditions may limit our comprehensive understanding of the distinct neural substrates of IC in MDD and PUD.

### Brain activation abnormalities in MDD and PUD versus HC

This study also found hyperactivation in the left vACC/mPFC and right temporal pole/STG in MDD relative to HC, which is consistent with a recent review reporting altered activation of these regions in Go/NoGo tasks in MDD (Piani et al., 2022a). The vACC/mPFC is a key node of the DMN (Andrews-Hanna et al., 2010; Li et al., 2023) (see Supplementary Table S1 for large-scale network mapping results), which has long been implicated in mind-wandering, rumination, and self-referential processing (Ferdek, van Rijn, & Wyczesany, 2016) and is deactivated in goal-oriented tasks (Smallwood et al., 2021). The right temporal pole/STG plays an important role in emotional processing and social cognition (Harada et al., 2018; Takahashi et al., 2010) and shows weakened connectivity with prefrontal executive regions (e.g. IFG) during response inhibition in MDD (Sheng et al., 2025). Hyperactivation in the left vACC/mPFC and right temporal pole/STG during IC in MDD may index difficulty in suppressing internally oriented thinking and enhanced emotional and social processing in response to task demands, which may interfere with task-focused control (Takahashi et al., 2010; Wang et al., 2023).

The overactivated left precentral gyrus and right precuneus during IC tasks in PUD relative to HC mainly overlapped with the DMN (Supplementary Table S1). The precentral gyrus is primarily responsible for voluntary motor control (Banker & Tadi, 2024), and the precuneus is integral to a broad array of higher-level cognitive functions, including self-reflection, mental imagery, and conscious awareness (Murray, Schaer, & Debbané, 2012). Therefore, hyperactivation in these regions may reflect enhanced motor reactivity and dysregulated self-related processing during goal-directed tasks in PUD. Notably, most of the findings were present only for cocaine users in terms of subgroup meta-analysis, possibly due to the limited statistical power to detect significant effects in methamphetamine users, given the relatively small sample size.

### Effects of demographic and clinical variables

Meta-regression indicated that MDD studies with a higher proportion of women showed increased brain activation in the right MFG, which aligns with previous findings that females showed greater activation in bilateral MFG in Go/NoGo tasks (Garavan et al., 2006). This correlation may be partly attributed to the higher female ratio in MDD. In PUD, older patients showed less activation in the right SFG relative to younger ones, implying that they may have more pronounced dysfunction in executive control (Cabeza & Dennis, 2013). We also observed a modulatory effect of duration of psychostimulant use in the left IPL. The long-term abuse of a psychostimulant can disrupt dopaminergic pathways, which may impair the function of attention and cognitive control networks (Smith et al., 2014). Moreover, longer abstinence was associated with increased activation in the left MFG, suggesting a potential recovery of top-down control systems that are typically impaired in active psychostimulant users (Le et al., 2021). Notably, although comorbidity had modulatory effects on PUD, which may indicate that co-occurring psychiatric conditions may exacerbate impairments in cognitive control and attentional processing in individuals with PUD (Miguel et al., 2023), further control analyses suggested no comorbidity effects on common and distinct activation abnormalities in MDD and PUD.

### Limitations of this study

Several important caveats should be taken into account when interpreting the results of this study. First, the meta-analyses depend on peak coordinates extracted from individual studies. Incorporating original statistical maps, combined with more original studies, will increase the power to detect smaller but meaningful results in future studies. Second, the representativeness of our meta-analysis results may be limited due to study heterogeneity in factors such as gender ratio, task paradigm, comorbidity, cocaine or methamphetamine users, and abstinence length. Although we accounted for these confounding factors via meta-regression or subgroup analyses, future meta-analyses matching the demographics and clinical conditions of these disorders might be of great interest when more studies emerge. Third, although we employed a series of control analyses to explore the potential impact of comorbidity, some of the original studies may not have screened for comorbidity with another, and subclinical symptoms (such as depression or anxiety) may have been present, particularly in the PUD group. This may confound the interpretation of the disorder-differential findings and warrants further investigation. Finally, we were unable to delineate the neural systems of cognitive inhibition or compare them with those of response inhibition due to the limited number of studies, especially in MDD, which requires further research to address this issue.

## Conclusion

This meta-analysis, to the best of our knowledge, is the first to focus on the common and distinct neurofunctional mechanisms of IC in MDD and PUD. The convergent alteration in the right IFG/MFG consolidates its role as a transdiagnostic marker of IC across psychiatric disorders, and the disorder-differentiating neurofunctional dysregulations in MCG in MDD and in bilateral IPL in PUD point to distinct mechanisms underlying shared IC deficits in MDD and PUD. Our findings may have implications for the development of novel intervention strategies to enhance IC in MDD and PUD by precisely matching the therapeutic targets.

## Supporting information

Li et al. supplementary material Li et al. supplementary material

## Data Availability

Coordinates and t-value files are available at https://osf.io/9dxkn/.

## Supplementary material

The supplementary material for this article can be found at http://doi.org/10.1017/S0033291725101141.
