# Supplementary material for: Common and distinct patterns of brain activity alterations during inhibitory control in depression and psychostimulant users: a comparative meta-analysis of task-based fMRI studies
Source: Psychol Med. 2025 Jul 28;55:e218. doi: 10.1017/S0033291725101141 (PMC12315649; doi:10.1017/S0033291725101141)
Supplement: Li et al. supplementary material [file S0033291725101141sup001.docx]

**Supplementary Materials**

1. **Supplementary Methods**

**1.1 Literature search and selection**

Database search keywords included: (("response inhibition"), OR ("inhibitory control"), OR ("interference"), OR ("action withholding"), OR ("action cancellation"), OR ("stop signal"), OR ("stopping"), OR ("go nogo"), OR ("action restraint"), OR ("countermanding"), OR ("inhibition"), OR ("stroop"), OR ("executive function"), OR ("flanker"), OR ("antisaccade"), OR ("cognitive inhibition"), OR ("cognitive control")) AND ((("MRI"), OR ("magnetic resonance imaging"), OR ("fMRI"), OR ("functional MRI")) AND (("depression"), OR ("depressive disorder"), OR ("MDD")) OR (("cocaine"), OR ("methamphetamine"), OR ("psychostimulant"), OR ("stimulant"))

**1.2 Controlling for comorbidity effects**

***Comparative meta-analyses.*** To address possible confounding effects of comorbidity on the group differences observed in comparative meta-analyses, we repeated the analyses incorporating comorbidity ratio as a covariate together with age and gender ratio. We applied default parameters and thresholds (FWHM = 20 mm, voxel-wise *p* < 0.005, SDM-Z > 1, and cluster extent size ≥ 10 voxels).

***Conjunction/disjunction meta-analyses.*** We excluded studies with comorbid patients from conjunction/disjunction analyses to control for comorbidity effects on identification of shared and disjunctive brain activation abnormalities between major depressive disorder (MDD) and psychostimulant use disorder (PUD). This exclusion method offered a comparably stringent control for comorbidity, given the lack of covariate options in the conjunction/disjunction model of SDM. A total of 8 MDD datasets from 7 studies (n = 143) and 8 PUD datasets from 8 studies (n = 193) of non-comorbid patients were included in the conjunction/disjunction analyses. The threshold was set at *p* < 0.0025 and cluster extent size ≥ 10 voxels.

**2. Supplementary Results**

**2.1 Subgroup analyses**

***Unmedicated MDD (Table S4).*** Compared to healthy controls (HC, n = 196, from 8 datasets of 8 studies), unmedicated MDD patients (n = 251, from 11 datasets of 8 studies) showed hyperactivation in the right inferior parietal lobule (IPL)/angular gyrus (AG), left IPL and right temporal pole/superior temporal gyrus (STG), and hypoactivation in the median cingulate/paracingulate gyri (MCG), right superior occipital gyrus (SOG), right middle frontal gyrus (MFG) and left parahippocampal gyrus (PHG). This analysis was not conducted for PUD, where most studies did not report on medication.

***Cocaine users (Table S5).*** Compared to HC (n = 210, from 8 datasets of 8 studies), cocaine users (n = 203, from 9 datasets of 8 studies) showed hyperactivation in the left fusiform gyrus, right SFG, right inferior occipital gyrus (IOG), left precentral gyrus and right supplementary motor area (SMA), and hypoactivation in the right IPL, left IPL/AG and right MFG.

***Methamphetamine users (Table S5).*** Compared to HC (n = 63, from 3 datasets of 3 studies), methamphetamine users (n = 55, from 3 datasets of 3 studies) showed hyperactivation in the left calcarine fissure and surrounding cortex, left inferior parietal gyrus (IFG) and left inferior temporal gyrus (ITG), and hypoactivation in the right postcentral gyrus, right IPL/AG, right SOG, right IFG and right ITG.

***Response inhibition tasks (Table S6).*** Compared to HC (n = 285, from 13 datasets of 13 MDD studies), MDD patients (n = 322, from 16 datasets of 13 studies) showed hyperactivation in the right IPL/supramarginal gyrus (SMG), left IPL, left ventral anterior cingulate cortex/medial prefrontal cortex (vACC/mPFC) and right temporal pole/STG and hypoactivation in the MCG. Compared to HC (n = 179, from 6 datasets of 6 PUD studies), PUD patients (n = 171, from 7 datasets of 6 studies) showed hyperactivation in the left precentral gyrus, left fusiform gyrus (FG), right inferior occipital gyrus (IOG), right SMA and right precuneus, and hypoactivation in the left IPL/AG, right IPL and right MFG.

***Cognitive inhibition tasks (Table S6).*** Compared to HC (n = 94, from 5 datasets across 5 studies), PUD patients (n = 87, from 5 datasets across 5 studies) showed hyperactivation in the right precuneus and hypoactivation in the right SMG, right IFG, right middle temporal gyrus (MTG), right SOG and right precentral gyrus. This analysis was not conducted for MDD, where only one study used cognitive inhibition tasks.

**Table S1. Spatial overlap between Yeo 7 networks and the identified brain regions in MDD and PUD**

| **Regions** | **Number of voxels** | | | | | | | **Total voxel number** | **Percentage overlapping voxels** |
| --- | --- | --- | --- | --- | --- | --- | --- | --- | --- |
|  | **VN** | **SMN** | **DAN** | **SAN** | **LN** | **FPN** | **DMN** |  |  |
| **Comparison of MDD *vs* HC** | | | | | | | | | |
| L vACC/mPFC | 0 | 0 | 0 | 0 | 13 | 0 | 156 | 169 | 8% LN; 92% DMN |
| L IPL | 0 | 0 | 0 | 10 | 0 | 56 | 131 | 197 | 5%SAN; 28% FPN; 66% DMN |
| R TP/STG | 0 | 0 | 0 | 0 | 46 | 0 | 19 | 65 | 71% LN; 29% DMN |
| R IPL | 0 | 0 | 0 | 1 | 0 | 5 | 53 | 59 | 2% SAN; 8% FPN; 90% DMN |
| L FG | 0 | 0 | 4 | 0 | 0 | 0 | 0 | 4 | 100% FG |
| MCG | 0 | 193 | 0 | 366 | 0 | 604 | 81 | 1244 | 16% SMN; 29% SAN; 49% FPN; 26% DMN |
| R IFG | 0 | 0 | 3 | 0 | 0 | 453 | 159 | 615 | 74% FPN; 26% DMN |
| **Comparison of PUD *vs* HC** | | | | | | | | | |
| L precentral | 0 | 1 | 183 | 0 | 0 | 0 | 271 | 455 | 40% DAN; 60% DMN |
| R precuneus | 0 | 0 | 0 | 0 | 0 | 7 | 187 | 194 | 4% FPN; 96% DMN |
| L FG | 14 | 0 | 0 | 0 | 0 | 0 | 0 | 14 | 100% VN |
| R SFG | 0 | 0 | 0 | 0 | 0 | 7 | 3 | 10 | 70% FPN; 30% DMN |
| R IPL | 0 | 0 | 277 | 31 | 0 | 839 | 387 | 1534 | 18% DAN; 2%SAN; 55% FPN; 25% DMN |
| L IPL/AG | 7 | 0 | 19 | 81 | 0 | 158 | 891 | 1156 | 1% VN; 2% DAN; 7% SAN; 14% FPN; 77% DMN |
| R MFG | 0 | 0 | 0 | 0 | 0 | 30 | 0 | 30 | 100% FPN |
| R MFG | 0 | 0 | 0 | 0 | 0 | 18 | 0 | 18 | 100% FPN |
| **Comparison of MDD *vs* PUD** | | | | | | | | | |
| R IPL | 0 | 0 | 464 | 173 | 0 | 1155 | 610 | 2402 | 19% DAN, 7%SAN; 48% FPN; 25% DMN |
| L IPL | 0 | 0 | 43 | 205 | 0 | 339 | 877 | 1464 | 3% DAN; 14%SAN; 23% FPN; 60% DMN |
| L IPL | 0 | 0 | 0 | 0 | 0 | 0 | 10 | 10 | 100% DMN |
| MCG | 0 | 33 | 0 | 252 | 0 | 662 | 642 | 1589 | 2% SMN; 16% SAN; 42% FPN; 40% DMN |
| L Parahippocampal | 64 | 0 | 0 | 0 | 0 | 0 | 0 | 64 | 100% VN |
| L MFG | 0 | 0 | 11 | 0 | 0 | 0 | 95 | 106 | 10% DAN; 90% DMN |
| L ITG | 0 | 0 | 0 | 0 | 11 | 0 | 0 | 11 | 100% LN |
| **Conjunction/disjunction analysis** | | | | | | | | |  |
| *MDD hypoactivation + PUD hypoactivation* | | | | | | | | | |
| R IFG/MFG | 0 | 0 | 0 | 0 | 0 | 580 | 390 | 970 | 60% FPN, 40% DMN |
| *MDD hyperactivation + PUD hyperactivation* | | | | | | | | | |
| R IPL | 0 | 0 | 90 | 114 | 0 | 766 | 655 | 1625 | 6%DAN, 7%SAN, 47%FPN, 40%DMN |
| L IPL | 0 | 0 | 75 | 203 | 0 | 342 | 760 | 1380 | 5% DAN, 15% SAN, 25% FPN, 55%DMN |
| *MDD hypoactivation +PUD hyperactivation* | | | | | | | | | |
| MCG | 0 | 0 | 0 | 44 | 0 | 274 | 242 | 560 | 8% SAN, 49% FPN, 43% DMN |

Abbreviations: AG, angular gyrus; DAN, dorsal attention network; DMN, default mode network; FG, fusiform gyrus; FPN, frontoparietal network; HC, healthy control; IFG, inferior frontal gyrus; IPL, inferior parietal lobule; ITG, inferior temporal gyrus; L, left; MCG, median cingulate/paracingulate gyri; LN, limbic network; MDD, major depressive disorder; MFG, middle frontal gyrus; mPFC, medial prefrontal cortex; R, right; SAN, salience network; SFG, superior frontal gyrus; SMN, sensorimotor network; STG, superior temporal gyrus; TP, temporal pole; vACC, ventral anterior cingulate cortex; VN, visual network.

**Table S2. Brain regions significantly associated with demographic and clinical variables**

| **MNI coordinates** | **SDM-Z** | **Voxels** | **Regions (*p* < 0.0005, cluster size ≥ 20 voxels)** | **BA** |
| --- | --- | --- | --- | --- |
| **MDD** | | | |  |
| **Gender ratio** |  |  |  |  |
| 2, 52, -2 | 1.138 | 74 | Right middle frontal gyrus, orbital part | \ |
| -16, 22, 4 | 1.083 | 22 | Left caudate nucleus | \ |
| **PUD** |  |  |  |  |
| **Mean age** |  |  |  |  |
| -10, 56, 14 | -3.396 | 653 | Right superior frontal gyrus | \ |
| 42, 10, 2 | 2.140 | 362 | Right insula | 48 |
| **Comorbidity ratio** |  |  |  |  |
| 50, -52, 46 | 1.093 | 985 | Right inferior parietal lobule | 40 |
| -4, 58, 20 | -5.232 | 151 | Left superior frontal gyrus | 10 |
| **Duration** |  |  |  |  |
| -56, -44, 48 | -3.213 | 23 | Left inferior parietal lobule | 40 |
| **Abstinence days** |  |  |  |  |
| -14, 56, 26 | -1.479 | 1002 | Left superior frontal gyrus | 10 |
| -48, 12, 40 | 3.332 | 229 | Left middle frontal gyrus | 44 |

Abbreviations: BA, Brodmann areas; MDD, major depressive disorder; PUD, psychostimulant use disorder; SDM, seed-based d mapping.

**Table S3. Controlling for comorbidity effects in comparative meta-analysis and conjunction/disjunction analysis**

| **MNI coordinates** | **SDM-Z** | **Voxels** | **Regions** | **BA** |
| --- | --- | --- | --- | --- |
| **Comparative meta-analysis^a^** | | |  |  |
| **MDD > PUD** |  |  |  |  |
| 50, -60, 48 | 2.510 | 2531 | Right inferior parietal lobule/ angular gyrus | 39 |
| -50, -50, 42 | 2.499 | 1373 | Left inferior parietal lobule | 40 |
| -36, -78, 46 | 1.458 | 28 | Left inferior parietal lobule | \ |
| **MDD < PUD** |  |  |  |  |
| 0, -26, 34 | -2.372 | 2232 | Median cingulate/paracingulate gyri | 23 |
| -46, 12, 44 | -1.376 | 120 | Left middle frontal gyrus | 9 |
| -34, -46, -4 | -1.510 | 110 | Left parahippocampal gyrus | \ |
| -54, -24, -24 | -1.153 | 54 | Left inferior temporal gyrus | 20 |
| 2, 24, 18 | -1.131 | 34 | Right anterior cingulate/paracingulate gyri | \ |
| 0, 22, 30 | -1.089 | 18 | Left anterior cingulate/paracingulate gyri | \ |
| **Conjunction/Disjunction analysis^b^** | | | | |
| Convergent hypoactivation in MDD and PUD | | | |  |
| 46, 44, 4 | \ | 1247 | Right inferior/middle frontal gyrus | 45 |
| Hyperactivation in MDD but hypoactivation in PUD | | | |  |
| 62, -40, 38 | \ | 2066 | Right inferior parietal lobule/supramarginal gyrus | 40 |
| -50, -48, 38 | \ | 1725 | Left inferior parietal gyrus | 40 |

Abbreviations: BA, Brodmann areas; HC, healthy control; MDD, major depressive disorder; PUD: psychostimulant use disorder; SDM, seed-based d mapping. a, voxel-wise threshold *p* < 0.005, cluster size ≥ 10 voxels. b, voxel-wise threshold *p* < 0.0025, cluster size ≥ 10 voxels.

**Table S4. Subgroup-analysis results in unmedicated MDD**

| **MNI coordinates** | **SDM-Z** | **Voxels** | **Regions (voxel-wise threshold p < 0.005, cluster size ≥ 10 voxels)** | **BA** |
| --- | --- | --- | --- | --- |
| **Comparison of unmedicated MDD *vs* HC** | | |  |  |
| **MDD > HC** |  |  |  |  |
| 46, -62, 46 | 1.516 | 1432 | Right inferior parietal lobule/ angular gyrus | 39 |
| -50, -52, 40 | 1.503 | 529 | Left inferior parietal lobule | 40 |
| 34, 14, -24 | 1.160 | 138 | Right temporal pole/superior temporal gyrus | \ |
| **MDD < HC** |  |  |  |  |
| 0, -26, 36 | -2.124 | 1892 | Median cingulate/paracingulate gyri | 23 |
| 22, -94, 16 | -1.022 | 96 | Right superior occipital gyrus | 18 |
| 44, 44, 16 | -1.023 | 45 | Right middle frontal gyrus | 45 |
| -32, -42, -8 | -1.021 | 30 | Left parahippocampal gyrus | 37 |

Abbreviations: BA, Brodmann areas; HC, healthy control; MDD, major depressive disorder; SDM, seed-based d mapping.

**Table S5. Subgroup-analysis results in cocaine and methamphetamine users**

| **MNI coordinates** | **SDM-Z** | **Voxels** | **Regions (voxel-wise threshold p < 0.005, cluster size ≥ 10 voxels)** | **BA** |
| --- | --- | --- | --- | --- |
| **Cocaine users > HC** |  |  |  |  |
| -32, -70, -8 | 1.347 | 164 | Left fusiform gyrus | \ |
| 18, 46, 28 | 1.358 | 109 | Right superior frontal gyrus, dorsolateral | \ |
| 32, -76, -6 | 1.137 | 56 | Right inferior occipital gyrus | \ |
| -44, -2, 36 | 1.181 | 49 | Left precentral gyrus | \ |
| -34, -52, -4 | 1.131 | 38 | \ | \ |
| -50, 10, 48 | 1.181 | 24 | Left precentral gyrus | 9 |
| 26, -62, 20 | 1.071 | 11 | \ | 8 |
| 10, 18, 48 | 1.055 | 10 | Right supplementary motor area | \ |
| **Cocaine users < HC** |  |  |  |  |
| 50, -56, 44 | -2.931 | 1323 | Right inferior parietal lobule | 40 |
| -44, -70, 38 | -3.215 | 1304 | Left inferior parietal lobule/angular gyrus | 19 |
| 42, 48, 0 | -2.123 | 52 | Right middle frontal gyrus | 46 |
| 36, 58, 10 | -2.133 | 34 | Right middle frontal gyrus | 10 |
| **Methamphetamine users > HC** | | | |  |
| -6, -70, 22 | 1.368 | 569 | Left calcarine fissure and surrounding cortex | \ |
| -52, 16, 34 | 1.368 | 219 | Left inferior frontal gyrus, opercular part | \ |
| -60, -34, -18 | 1.367 | 216 | Left inferior temporal gyrus | 20 |
| **Methamphetamine users < HC** | | | |  |
| 58, -20, 36 | -1.211 | 534 | Right postcentral gyrus | 43 |
| 30, -66, 46 | -1.158 | 244 | Right inferior parietal gyrus/ angular gyrus | 7 |
| 20, -98, 20 | -1.266 | 86 | Right superior occipital gyrus | \ |
| 58, 26, 16 | -1.266 | 77 | Right inferior frontal gyrus, triangular part | 45 |
| 52, 4, 50 | -1.147 | 30 | Right precentral gyrus | 6 |
| 42, -52, -14 | -1.092 | 24 | Right inferior temporal gyrus | 37 |
| 48, 22, -6 | -1.107 | 23 | Right inferior frontal gyrus, orbital part | 38 |
| 50, -56, -6 | -1.098 | 11 | Right inferior temporal gyrus | 37 |

Abbreviations: BA, Brodmann areas; HC, healthy control; SDM, seed-based d mapping.

**Table S6. Subgroup-analysis results in response inhibition and cognitive inhibition tasks**

| **MNI coordinates** | **SDM-Z** | **Voxels** | **Regions (voxel-wise threshold p < 0.005, cluster size ≥ 10 voxels)** | **BA** |
| --- | --- | --- | --- | --- |
| **Response inhibition tasks** | | | |  |
| **MDD > HC** |  |  |  |  |
| 48, -44, 44 | 1.566 | 960 | Right inferior parietal lobule/supramarginal gyrus | 40 |
| -48, -50, 42 | 1.371 | 212 | Left inferior parietal lobule | 40 |
| -6, 28, -8 | 1.470 | 170 | Left ventral anterior cingulate cortex/medial prefrontal cortex | 11 |
| 38, 18, -28 | 1.202 | 59 | Right temporal pole/superior temporal gyrus | 38 |
| **MDD < HC** |  |  |  |  |
| 0, -24, 32 | -1.913 | 1902 | Median cingulate/paracingulate gyri | \ |
| -36, -42, -8 | -1.023 | 31 | \ | \ |
| **PUD > HC** |  |  |  |  |
| -44, -2, 36 | 1.341 | 121 | Left precentral gyrus | \ |
| -30, -68, -10 | 1.045 | 71 | Left fusiform gyrus | \ |
| 34, -76, -6 | 1.045 | 69 | Right inferior occipital gyrus | \ |
| -32, -50, -6 | 1.045 | 67 | Left fusiform gyrus | 37 |
| -50, 10, 48 | 1.340 | 43 | Left precentral gyrus | 9 |
| 10, 18, 48 | 1.045 | 31 | Right supplementary motor area | \ |
| 16, -38, 6 | 1.045 | 26 | Right precuneus | \ |
| 26, -60, 20 | 1.045 | 20 | \ | \ |
| **PUD < HC** |  |  |  |  |
| -48, -70, 32 | -3.442 | 1187 | Left inferior parietal lobule/angular gyrus | 39 |
| 52, -58, 48 | -2.584 | 753 | Right inferior parietal lobule | \ |
| 42, 48, 4 | -2.189 | 58 | Right middle frontal gyrus | 46 |
| 38, 58, 10 | -2.187 | 39 | Right middle frontal gyrus |  |
| **Cognitive inhibition tasks** | | |  |  |
| **PUD > HC** |  |  |  |  |
| 6, -68, 36 | 1.786 | 389 | Right precuneus | 7 |
| **PUD < HC** |  |  |  |  |
| 58, -34, 36 | -1.586 | 2011 | Right supramarginal gyrus | 40 |
| 48, 22, -12 | -1.557 | 651 | Right inferior frontal gyrus, orbital part | 38 |
| 56, -46, 0 | -1.482 | 552 | Right middle temporal gyrus | \ |
| 58, 26, 18 | -1.229 | 44 | Right inferior frontal gyrus, triangular part | 45 |
| 20, -98, 20 | -1.213 | 29 | Right superior occipital gyrus | \ |
| 52, 4, 50 | -1.126 | 22 | Right precentral gyrus | 6 |

Abbreviations: BA, Brodmann areas; HC, healthy control; MDD, major depressive disorder; PUD, psychostimulant use disorder; SDM, seed-based d mapping.

**Table S7. Heterogeneity analysis**

| **MNI coordinates** | **SDM-Z** | **Voxels** | **Regions (voxel-wise threshold p < 0.005, cluster size ≥ 10 voxels)** | **BA** |
| --- | --- | --- | --- | --- |
| **MDD > HC** | | | |  |
| -20, -42, -26 | 2.431 | 674 | Left cerebellum | 37 |
| 46, -50, 46 | 3.149 | 350 | Right inferior parietal lobule | 40 |
| **PUD > HC** | | |  |  |
| -6, 58, 18 | 3.211 | 344 | Left superior frontal gyrus | 10 |
| 60, -18, 36 | 2.396 | 115 | Right postcentral gyrus | 43 |
| 0, -56, 28 | 2.349 | 89 | Precuneus | \ |
| 40, 12, 0 | 2.642 | 64 | Right insula | 48 |
| -44, 12, 42 | 2.725 | 54 | Left precentral gyrus | 44 |
| 48, 22, -4 | 2.437 | 38 | Right inferior frontal gyrus, orbital part | 47 |
| -54, -24, -24 | 2.316 | 28 | Left inferior temporal gyrus | 20 |
| 58, 26, 14 | 2.805 | 28 | Right inferior frontal gyrus, triangular part | 45 |
| 20, -98, 20 | 2.786 | 24 | Right superior occipital gyrus | \ |
| 30, -66, 46 | 2.064 | 14 | Right angular gyrus | 7 |

Abbreviations: BA, Brodmann areas; HC, healthy control; MDD, major depressive disorder; PUD, psychostimulant use disorder; SDM, seed-based d mapping
